# Comparative histology of muscle in free ranging cetaceans: shallow versus deep diving species

**DOI:** 10.1038/srep15909

**Published:** 2015-10-30

**Authors:** E. Sierra, A. Fernández, A. Espinosa de los Monteros, J. Díaz-Delgado, Y. Bernaldo de Quirós, N. García-Álvarez, M. Arbelo, P. Herráez

**Affiliations:** 1 Department of Veterinary Pathology, Institute for Animal Health, Veterinary School, University of Las Palmas de Gran Canaria, Spain.

## Abstract

Different marine mammal species exhibit a wide range of diving behaviour based on their breath-hold diving capabilities. They are classically categorized as long duration, deep-diving and short duration, shallow-diving species. These abilities are likely to be related to the muscle characteristics of each species. Despite the increasing number of publications on muscle profile in different cetacean species, very little information is currently available concerning the characteristics of other muscle components in these species. In this study, we examined skeletal muscle fiber type, fiber size (cross sectional area and lesser diameter), intramuscular substrates, and perimysium-related structures, by retrospective study in 146 stranded cetaceans involving 15 different species. Additionally, we investigated diving profile-specific histological features. Our results suggest that deep diving species have higher amount of intramyocyte lipid droplets, and evidence higher percentage of intramuscular adipose tissue, and larger fibre sizes in this group of animals.

Cetaceans exhibit a wide range of body masses and sizes^1^,^2^, though the gross distribution of skeletal muscle, particularly the epaxial and hypaxial components, appears to be similar in all species, providing a somewhat alike tail-stroke power for swimming. Moreover, they exhibit a wide range of diving behaviour based on their breath-hold diving capabilities and are these likely to be related to the muscle characteristics of each species^3^. According to the length and depth of dives, cetaceans, as other marine mammals (MMs), are categorized as long duration, deep-diving and short duration, shallow-diving species.

Fibre-type composition varies widely between muscles, in accordance with their functional requirements. Marine mammals that regularly perform deep, long-duration dives have locomotor muscles mainly composed of large type I fibres^4^ containing elevated myoglobin (Mb) concentrations. A novel myofiber profile for diving mammals, characterized by a muscle composition of ~80% fast-twitch (Type II) fibers with low mitochondrial volume densities, has been recently described in beaked whales^5^. Thus, there is a wide array of muscle adaptations for breath holding and apneic underwater diving.

The last decade has seen an increased interest regarding the locomotor muscle profile in different MM species^4^,^5^,^6^,^7^,^8^. However, very little information is currently available regarding the characteristics of other muscle components in cetaceans, such as tendons or perimysium-related structures^9^,^10^,^11^. The skeletal muscle is composed of slow (type I) and fast-twitch (type II) muscle fibres, which have high potential in aerobic and anaerobic adenosine triphosphate (ATP) production, respectively. Type II myofibers can be further subdivided according to their metabolic and physiologic features, by means of enzymohistochemical analysis.

The skeletal muscle can oxidize either carbohydrate or lipid to produce energy. Glucose is stored in the muscle as intramyocellular glycogen deposits, while lipids are accumulated within muscle in two distinct compartments: extramyocellular, stored in adipocytes residing in the interstitial connective tissue (ICT) or perimysium; and as intramyocellular lipid droplets (IMCL)^12^. The proportion of energy derived from each of these intramuscular resources is influenced by several factors: exercise type and duration and recruitment pattern of fiber types^13^,^14^.

The present study aimed to describe the histological features of the main epaxial locomotor muscle (*longissimus dorsi*) in free-ranging stranded cetaceans, with special emphasis on fiber type, fiber size (cross sectional area and lesser diameter), intramuscular substrates, and perimysium-related structures. Additionally, diving profile-specific histological features were investigated.

## Results

### Histological assessment

**Figure 1:**
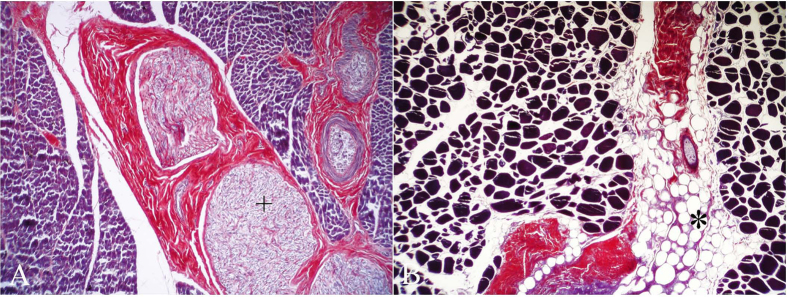
Interstitial connective tissue (perimysium). PTAH histochemical stain. (**A**) The red connective tissue was present in the interstitial spaces between muscle fibers and was observed in the areas intramuscular nerves (plus). Neonate male of striped dolphin (*Stenella coeruleoalba*). 100× magnification. (**B**) Variable fatty infiltration of the interstitial connective tissue was observed within the deep-diving species in our study. Juvenil female of Cuvier’s beaked whale (*Ziphius cavirostris*). 40× magnification.

The histological examination by HE showed that LD myofibres exhibited a homogeneous acidophilic staining and displayed a polygonal morphology with typical peripherally located nuclei. However, nuclei occupied an internal position at the myotendinous junctions. Tendons were composed of elongated fibroblasts scattered between dense parallel collagen bundles, being more easily appreciated with the PTAH technique (red), while the myofibers displayed an intense blue staining. Several specialized structures were noted within the supporting connective tissue, such as muscle spindles and intramuscular nerves (Fig. 1A).

Depending on the species, a variable amount of IAT intermingled with the perimysium was recognized, (Fig. 1B). Muscle spindles were evidenced as clusters of myofibers of small diameter within the connective tissue. Intramuscular nerve fibers varied slightly in diameter and number in the perimysium within the fascicle and among different fascicles and specimens. Although distinguishable by HE, they become more readily evident after PAS or PTAH techniques. Nerve fibres were surrounded by a dense connective tissue of lamellar arrangement, consisting of several concentric layers (perineurium).

The experiment conceived to evaluate autolysis progression at RT showed that skeletal muscle samples at T_0_ displayed the typical staining pattern and morphology by HE as described above. Concomitantly, myofibres showed a blue staining by PTAH and cross-striations could be identified in longitudinally-oriented tissue sections. By T_4_-T_5_ (56–68 h post-mortem) putrefaction gas-bubbles were evident. Myofiber nuclei disappeared by T_5_-T_6_ (68–80 h post-mortem). Likewise, samples exposed to freshwater showed that gas-bubbles appeared earlier, at T_3_ (44 h post-mortem), and that formalin pigment had built up in higher amounts than samples kept at RT. Blue myofibre staining pattern remained despite autolysis progression yet many of the myofibers failed to show the cross-striations by T_3_ and T_4_ at RT or underwater, respectively. The interstitial tissue componets remained recognisable, as cytoplasmic border remain distinct and collagen did not get dispersed and/or ragged, during all the experiment. Putrefaction bacteria were first noticed at T_3_.

**Figure 2:**
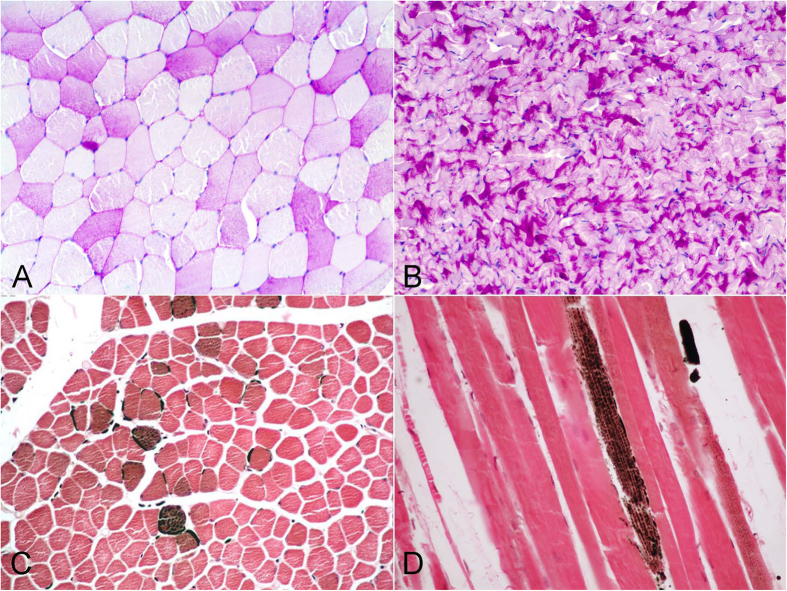
Intracellular substrates stores. (**A**) Intramyofiber glycogen. Adult male of pygmy sperm whale (*Kogia breviceps*). PAS stain, 100× magnification. (**B**) Intramyofiber glycogen. Neonate female of sperm whale (*Physeter macrocephalus*). PAS stain, 40× magnification. (**C**) Intramyofiber lipids were visualised as small droplets with a variable staining pattern ranging from olive green to black. Calf male of short-finned pilot whale (*Globicephala macrorhynchus*). Osmium tetroxide postfixation, 100× magnification. (**D**) Small fibres typically had a greater lipid concentration as revealed by OT. Adult female of Blainville’s beaked whale (*Mesoplodon densirostris*). Osmium tetroxide postfixation, 200× magnification.

Intramyofibre substrates were readily evident on tissue sections by means of the PAS technique (for glycogen), and the OT technique (for lipids). Glycogen was detected in 17/148 (11.5%) animals, and was visualised as uniformly distributed pink granules throughout the sarcoplasm of the myofibres in those animals with a very fresh/fresh decomposition code (Fig. 2A,B). In tissue sections having incipient autolysis, the glycogen was largely confined to the periphery of the myofibers.

**Figure 3:**
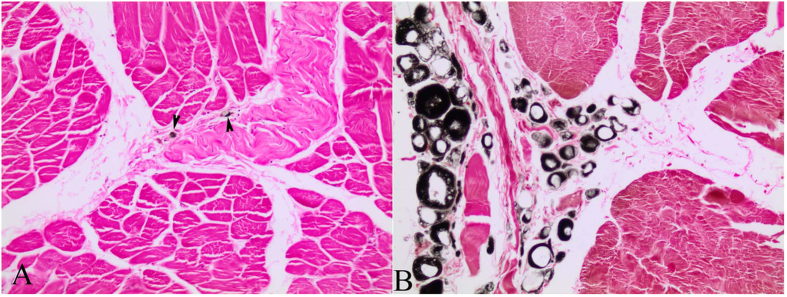
The occupied area of the interstitial adipose tissue varied greatly between species with different diving behaviours. Osmioum postfixation technique. Adipocytes were visualised as cells with a variable staining pattern ranging from olive green to black. (**A**) Adult female of bottlenose dolphin (*Tursiops truncatus*). Adipocytes were displayed within the interstitial connective tissue (arrowheads). 200× magnification. (**B**) High amount of adipocytes were displayed within the perimysium. Juvenile male of short-finned pilot whale (*Globicephala macrorhynchus*). 200× magnification.

Intramyofiber lipids were visualised as small droplets with a variable staining pattern ranging from olive green to black (Fig. 2C,D). Small fibres typically had a greater lipid concentration as revealed by OT. Lipids were visualized in 24/148 (16.2%) animals from 7 different cetacean species, the majority of which (95.8%) belonged to the deep-diving group (Table 1). The OT also stained the lipids contained in the interstitium as well as the myelin sheaths of the endoneurium. Variable fatty infiltration of the interstitial connective tissue was observed in the deep-diving group of cetaceans (mainly *Physeter macrocephalus*, *Globicephala macrorhynchus*, *Kogia breviceps* and *Ziphiidae* family) (Fig. 3).

### Immunohistochemical study

Immunolabelling with slow and fast MHC Abs, which recognize type I and II myofibers, respectively, revealed two clearly different myofiber types in all the analyzed species. The immunomarker revealed a diffuse and homogenous intracytoplasmic staining pattern for both, type I and type II myofibers. However, Mb immunolabelling varied according to the degree of autolysis of the tissue sections. Immunoexpression of Mb and fibrinogen in samples studied under both conditions, was not detectable at T_3_ and T_2_, respectively. Thus, IHC detection of Mb and fibrinogen in skeletal muscle samples is possible until 44 and 32 hours post-mortem, respectively.

### Electron microscopic study

**Figure 4:**
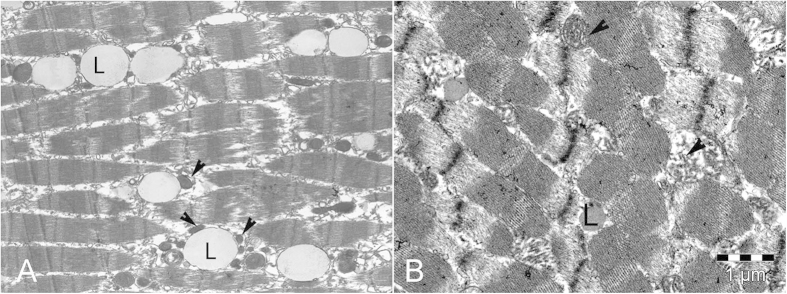
At the ultrastructural level, lipids (L) appeared as electrodense droplets within the myofibrils, closely related to the mitochondria (arrowheads), which were bigger and less electrodense in the deep-diving species. (**A**) Adult female of blainville’s beaked whale (*Mesoplodon densirostris*). (**B**) Adult specimen of Atlantic spotted dolphin (*Stenella frontalis*). Bar = 1 μm.

Ultrastructural analysis revealed lipids in both species examined regardless shallow (Atlantic spotted dolphin) and deep diving (blainville’s beaked whale) capabilities. Lipids appeared as electron-dense droplets within the myofibrils, closely related to the mitochondria. These lipid droplets were subjectively bigger and less electron-dense in deep-diving species (Fig. 4).

### Morphometric and Statistical studies

**Figure 5:**
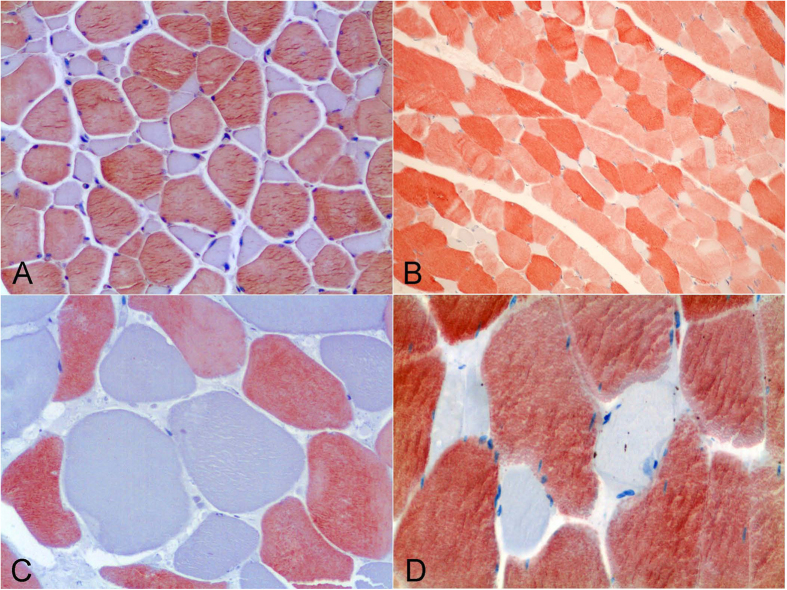
Fibre size comparison among species with different diving profile. Immunohistochemical stain for the fast MHC at light microscopy (200X magnification). (**A**) Adult female of common dolphin (*Delphinus delphis*). (**B**) Juvenile female of Atlantic spotted dolphin (*Stenella frontalis*). (**C**) Adult female of pygmy sperm whale (*Kogia breviceps*). (**D**) Adult female of Cuvier’s beaked whale (*Ziphius cavirostris*).

**Figure 6:**
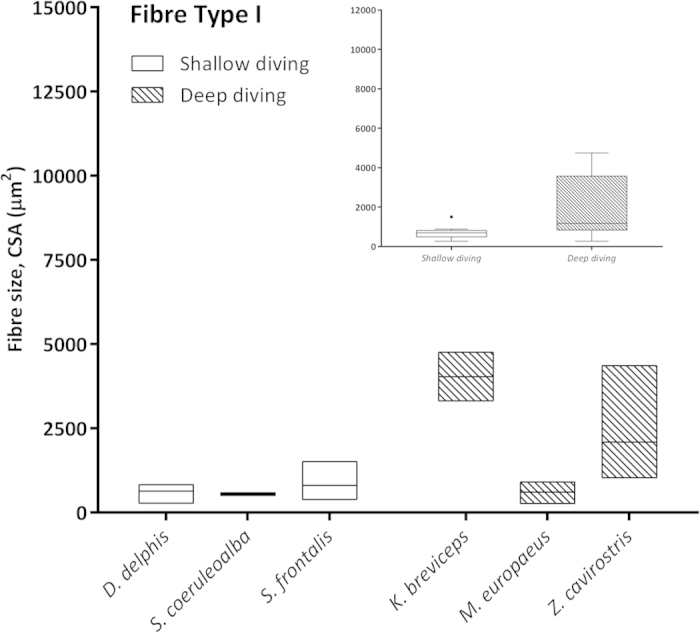
Mean fiber cross sectional area (CSA) (μm^2^) of Type I fibers in the shallow-diving group (*D. delphis, S. coeruleoalba, S. frontalis*) and deep-diving group (*K. breviceps, M. europaeus, Z. cavirostris*). Right inset: Global mean fiber CSA of Type I fibers were larger for the deep-diving group of animals.

**Figure 7:**
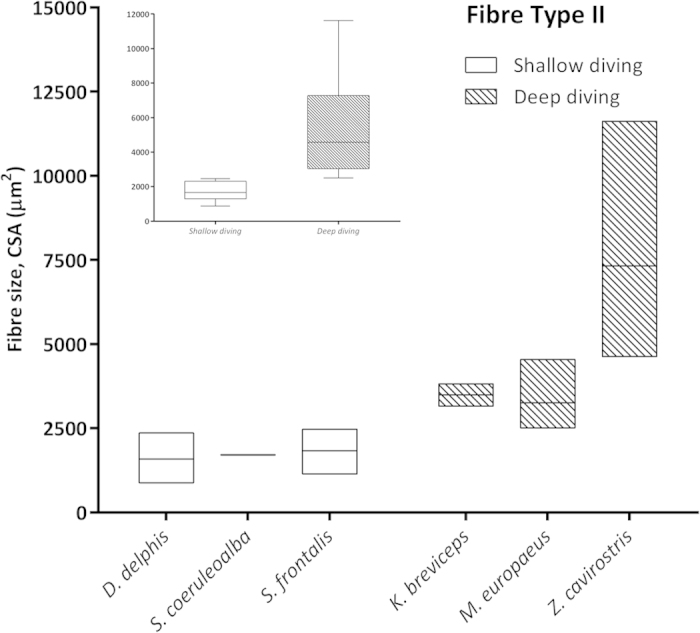
Mean fiber cross sectional area (μm^2^) of Type II fibers in the shallow-diving group (*D. delphis, S. coeruleoalba, S. frontalis*) and deep-diving group (*K. breviceps, M. europaeus, Z. cavirostris*). Left inset: Global mean fiber CSA of Type II fibers were larger for the deep-diving group of animals.

**Figure 8:**
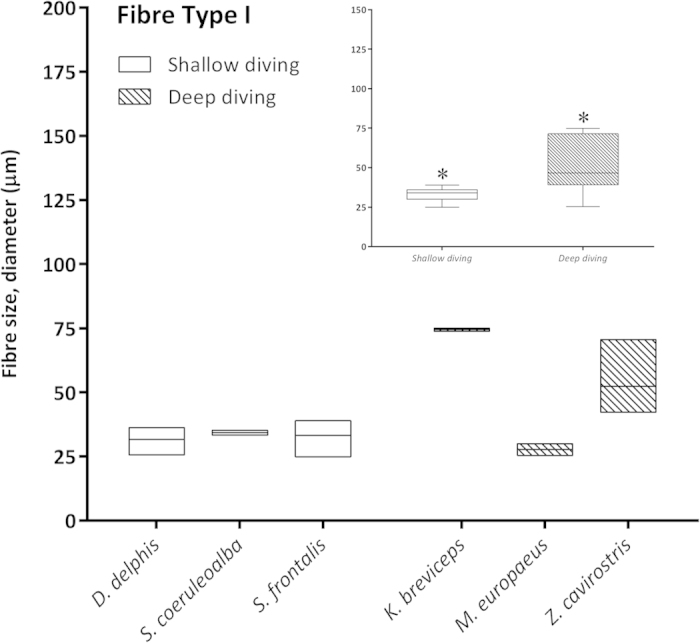
Mean fiber lesser diameter (LD) (μm) of Type I fibers in the shallow-diving group (*D. delphis, S. coeruleoalba, S. frontalis*) and deep-diving group (*K. breviceps, M. europaeus, Z. cavirostris*). Right inset: Global mean fiber LD of Type I fibers were larger for the deep-diving group of animals. Asterisks denote significant differences across groups (P < 0.05, the Mann-Whitney U test).

**Figure 9:**
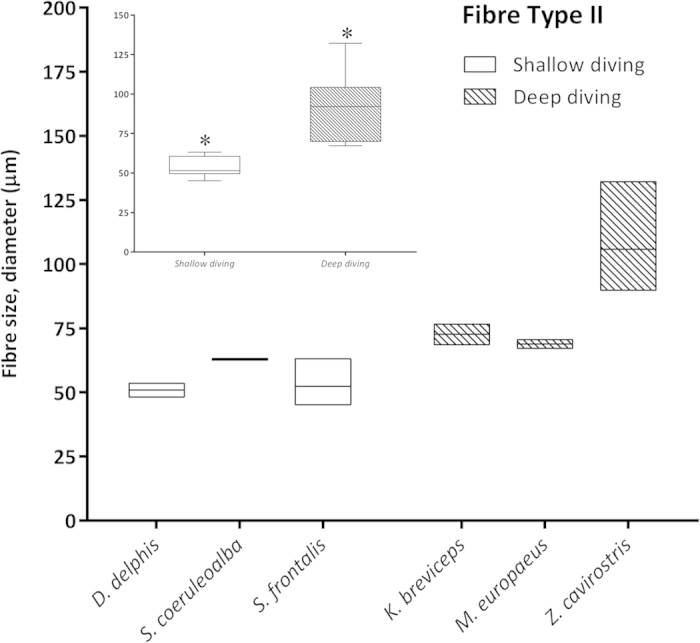
Mean fiber lesser diameter (LD) (μm) of Type II fibers in the shallow-diving group (*D. delphis, S. coeruleoalba, S. frontalis*) and deep-diving group (*K. breviceps, M. europaeus, Z. cavirostris*). Left inset: Global mean fiber LD of Type II fibers were larger for the deep-diving group of animals. Asterisks denote significant differences across groups (P < 0.05, the Mann-Whitney U test).

Morphometry performed on selected skeletal muscle tissue sections from both groups of animals (shallow- *versus* deep-diving profile) (Table 2) showed that larger myofiber sizes (CSA and LD) corresponded with fast-twitch myofibres in the majority of the analysed species (*D. delphis*, *S. coeruleoalba*, *S. frontalis*, *M. europaeus*, and *Z. cavirostris*). However, in the *K. breviceps* species, the Type I and II fibers were of similar diameter, with larger diameters corresponding to type I fibers. The largest myofiber sizes were found in adult Cuvier’s beaked whales, particularly the fast-twitch fibres [for both measured parameters; CSA (7,324.31 μm^2^) and LD (105.84) μm] (Fig. 5). Type II myofiber global mean diameter was approximately 1.5 times larger than Type I myofibers in both groups of animals (1.7 *versus* 1.6). However, this proportion was more stable within the shallow-diving group, while the *K. breviceps* and the *M. europaeus* species showed the extreme proportions (0.97 *versus* 2.52) within the deep-diving group (Table 2). The global mean fiber CSA and LD of both myofiber types were larger for the deep-diving group (Figs 6, 7, 8, 9). Statistical analysis of the myofiber size (LD) was performed on the shallow- and deep-diving groups. The Mann-Whitney U test for two independent-samples revealed a highly significant difference in the mean LD of the type I (P = 0.019) and type II (P = 0.000) myofibers when shallow- and deep-diving groups were compared. Thus, the hypothesis of the homogeneity of the mean values for the shallow- and deep-diving groups can be rejected at the 0.05% significance level.

**Figure 10:**
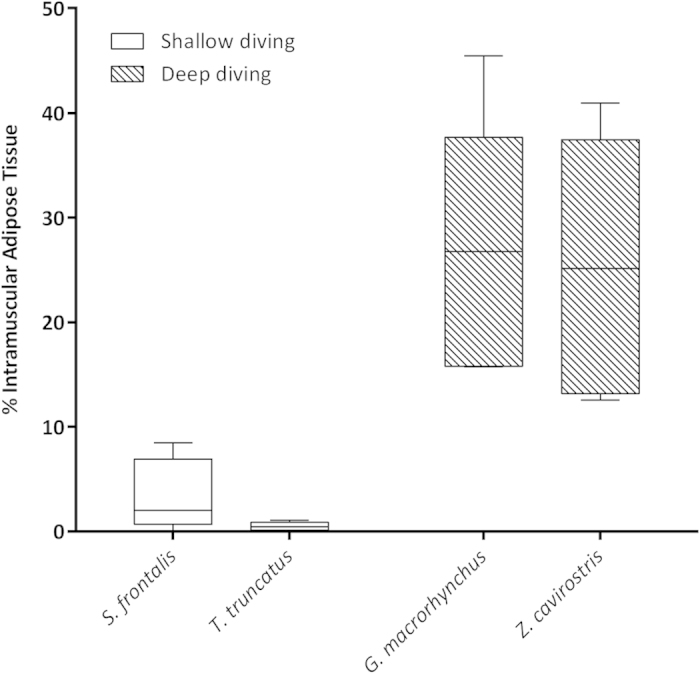
The occupied area by the adipocytes intermixed within the perimysium (IAT) was higher in the species from the deep-diving group. The morphometric analysis showed that the mean of the percentage area of the IAT in the shallow-diving group of animals was 1.95% *versus* 26.01% in the deep-diving group. Significant differences were found across groups (P < 0.05, the Mann-Whitney U test).

The area occupied by the adipocytes intermixed within the perimysium was higher in the deep-diving group. The morphometric analysis showed that the mean of the percentage area of the IAT in the shallow-diving group was 1.95% *versus* 26.01% in the deep-diving group (Table 3). A statistically significant (P = 0.000) difference was obtained when both groups were compared by the Mann-Whitney U test for two independent-samples (Fig. 10). Similar mean values were observed between species from the deep-diving group (26.74 for the short-finned pilot whales *versus* 25.29 for the Cuvier’s beaked whales), while the mean values for the Atlantic spotted dolphins and Atlantic bottlenose dolphins were quite dissimilar (3.44 *versus* 0.47) in the shallow-diving group.

## Discussion

This study describes the histological features of the LD muscle in free-ranging stranded cetaceans, with special emphasis on fiber size, intramuscular substrates, and perimysium-related structures, comparing them across species with different dive behaviours.

The microscopic fibre appearance, as polygonal well-delimited cells, corresponds with previous descriptions of skeletal muscle cells in land mammals, including the peripheral position of nuclei near the myotendinous junction^15^. It has been described that the amount, composition and structure of intramuscular connective tissue vary tremendously between muscles, species, breeds, and with animal age^16^. In our study, muscle spindles and intramuscular nerves were commonly structures observed within the perimysium of the analyzed samples. Muscle spindles are stretch receptors which are distributed within muscles and perceive its length and the velocity of muscle length change^17^. They consist of a few short and thin muscle fibres, called intrafusal fibres, wrapped by a capsule derived from terminal Schwann cells of sensory axons. Similar descriptions were observed in our study and, previously, in bottlenose dolphins^9^. In cetaceans, the axial musculature is innervated by the spinal nerves^18^,^19^. Intramuscular nerves, containing myelinated nerve fibres, were present in the LD samples from our study. The function of skeletal muscle is intimately related to the function of the peripheral nervous system. The physiologic attributes of a muscle fibre-its rate of contraction and type of metabolism (oxidative, anaerobic, or mixed)-are determined not only by the muscle cell itself but by the neuron responsible for its innervation, the ventral horn or the brain stem motor neurons^15^.

Differences in fiber type composition among MMs have been proposed to result from differences in routine dive duration and swim speed^20^. In accordance, it is generally accepted that locomotor muscles of deep-diving MMs share a suite of adaptations to prolong dive duration, i.e. elevated myoglobin concentrations and predominance of large Type I fibers. However, novel myofiber profiles have been recently described in extreme deep-diving whales (short-finned pilot- and beaked- whales)^5^. Beaked whales possessed a sprinter’s fiber-type profile, composed of ~80% Type II fibers, while in the short-finned pilot whales approximately 1/3 of the muscle fibers were an intermediate type of type I fibers, slow-twitch, oxidative, glycolytic fibers (SOG), demonstrating that muscle adaptations for underwater exercise are complex and diverse. Despite these differences, muscle of both cetaceans displayed high myoglobin concentrations and large fibers.

The quantitative morphometrical analysis of fibre size in the present study in two groups with different diving behaviour showed that larger areas for both fibre types corresponded to the long duration deep-diving group of cetaceans. The biggests size (CSA and LD) was for the type II fibres in the adult specimens from the Cuvier’s beaked whale’s species in our study. Similar results have been previously reported^5^, showing that the type II fibres of beaked whales fall within the size range of muscle fibres previously observed in other deep-diving mammal. In that study, type II fibres size (minor diameter = ~80 μm) from three different beaked whales’ species (*M. densirostris*, *M. europaeus,* and *M. mirus*) were included. However, similar fast-twitch fibre size to those of the genus *Mesoplodon* has been formerly reported for the *K. breviceps* species (minor diameter = 81,7 μm)^4^. In our study, type II LD for both species showed similar value (72.62 μm in the *K. breviceps versus* 73.38 μm in the *M. europaeus*). The difference in fiber size observed between these studies and the current one could be due to that different conservative methods were employed, frozen *versus* formalin-fixed paraffin-embedded (FFPE), for morphometry. The previous and current fiber size recorded for the genus *Mesoplodon* and the *K. breviceps* species, although large, are quite far from those of the Type II fibres displayed by the *Z. cavirostris* species recorded in our study, which are until now, the biggest sizes reported for a skeletal muscle fibre in a marine mammal’s specie (LD = 105.84 μm). The beaked whales, Cuvier’s beaked whale in particular, have exceptional diving capabilities. Recently, depths of more than 2900 m and dive durations of over 2 hours have been recorded in Cuviers’ beaked whale during single breath-hold dives^21^.

In mammals, aerobic Type I fibers tend to have smaller diameters than Type II fibers. In our study, this tendency was displayed for all the analysed specimens, with Type II fibers showing a fiber size relation (II:I) of approximately 1.55, except for the pygmy sperm whale species, which display slightly larger type I in the adult specimens (II:I = 0.97), representing the bigger size for type I fibres among all the analyzed species. Similar results have been previously described in this species and other long-duration, deep-diving species, the narwhal (*Monodon monoceros*)^4^,^22^.

The fiber type metabolic profile is not currently well characterized for marine mammals, but it is assumed that Type I fibers are oxidative^4^,^5^, although a slow-twitch fiber phenotype with both high oxidative and high glycolytic capacities (SOG) has been recently described in the short-finned pilot whale species^5^. However, this fiber type is unusual both in marine and land mammals^23^. An inverse relationship between skeletal muscle fiber size and its oxidative capacity has been described^24^. This assumption has been confirmed in the pygmy sperm whale, since this species has larger type I fibers and displayed a significantly lower index of mitochondrial density than a shallow-diving species (*T. truncatus*) across both fiber types did. Low indices of mitochondrial densities reduce the rate of oxygen consumption and have been proposed as an adaptive mechanism extending the aerobic dive limit in MMs^8^. In addition, concerning fiber size, it appears that large muscle fibres have a decreased basal metabolic cost compared with smaller ones, a fact that greatly contributes to reduce the overall metabolic rate^25^, which could be interpreted as another adaptation to increase underwater time of immersion in MMs.

Elevated myoglobin concentration, as one of the most important adaptation for breath hold diving^26^, has been proposed to offset low mitochondrial volume in deep-diving MMs, providing enhanced oxygen storage capacity. Myoglobin acts as the primary oxygen carrier in the skeletal muscles and its elevated Mb contents enables aerobic metabolism to be maintained during the diving apnea (breath-holding) of these mammals. We used a histological approach and immunoexpression to describe the presence of fiber Mb. Our results showed that type I and type II fibers expressed myoglobin in all the analysed species. Similar results have been previously reported by the locomotor muscle of beaked whales^27^, demonstrating that both type of muscle fibres act as a site of oxygen storage within the muscle in these species. However, oxidative metabolism in mitochondria not only depends on an adequate supply of oxygen, but also on substrates^28^. The skeletal muscle substrates supply involve intermediate stores both organismics (liver for glucose and adipose tissue for fatty acids) and intracellular (glycogen and lipid droplets inside myofibers)^28^. Myofibers require a great deal of energy in the form of adenosine triphosphate (ATP) to generate force and movement. Tipically, Type I fibers are oxidative and use lipids for ATP production, Type IIx/b fibers use glucose for anaerobic ATP production, and Type IIa fibers are probably oxidative but with an enhanced anaerobic capacity. In MMs, a heavy reliance of lipid aerobic energy metabolism during most dives has been described in seal muscle^3^,^8^,^29^ and in the Type I fibers of beaked whales^5^.

The PAS stain reflects glycogen content and crudely approximates fibre type distribution^30^. However, in our study, the presence of intramyofibre glycogen was influenced by the post-mortem interval. After death, glycogen decomposes rapidly^31^. Muscle cells can still use glycogen as an energy source to maintain an unstretched state before rigor mortis setting, but because the lack of oxygen, the citric acid cycle and the oxidative phosphorilation pathway no longer function. Marine wildlife animal studies rely upon stranded specimens with different post-mortem intervals, which invariably affect intracellular glycogen depots. In a similar way, lipid storage was not constantly displayed in the animals of our study, since several factors^32^ (endurance exercise, diet, autolysis) can deplenish them. In our study, only 16.4% of the specimens displayed IMCL with the OT, although the majority of them belong to species that routinely perform deep dives. Thus, the results of the current study suggest that swimming skeletal muscle of deep, long-duration diving species shows a greater amount of intramyocellular lipids droplets than swimming skeletal muscle of short-duration diving species, and that they were predominantly located in the fibres with minor diameter (interpreted as slow-twitch fibres). Lipids serve as a readily available source of high energy metabolic fuel for oxidative fibres, so lipid content is greater in slow than in fast fibers^9^,^33^.

This finding could be interpreted as an adaptation to prolong aerobic lipid-based metabolism under hypoxic dives, as it has been previously suggested in other deep-diving MMs^5^,^8^. Lipid droplets, as a source of energy for muscle, can potentially provide enough energy to support several hours or more of muscle metabolism at routine levels of exertion during aerobic dives in MMs^20^. In accordance, larger intramyocellular lipid stores have been observed in “athletic” species as well as in endurance-trained human athletes^34^.

The primary source of lipids for muscle metabolism are fatty acids and triglycerides that are transported in the plasma, from the subcutaneous and deep visceral adipose tissue, or triglycerides that are stored as lipid droplets (IMCL) between mitocondria and myofibrils^20^. Substrate stores are replenished at low flux rates during periods of rest and are stored intracellularly. Intramuscular fat represents the amount of fat accumulated between muscle fibers or within muscle cells. The close anatomic contact between intramuscular fat, in the form of IAT, and muscle cells suggests a reciprocal influence. In our study, the IAT occupied larger relative areas in muscle samples of deep-diving species (short-finned pilot whales and Cuvier’s beaked whales) than in those of shallow-diving species (Atlantic spotted dolphin and bottlenose dolphin). These results, in combination with the higher IMCL present in deep-diving species in our study, could represent that intramyocellular lipid stores can be replenished fast and easy from this nearly located source of triglycerides in this group of cetaceans according to their metabolic aerobic requeriments.

At the ultrastructural level, intramyofibrillar lipid droplets were present in specimens with different diving capabilities, although lipid size and density were different among the two groups. Such differences could reflect interspecific variability in the chemical composition of intramyofibrilar lipids, as it has been formerly suggested^9^. In addition, it has been previously suggested that, in dogs, the size of intracelular substrate pools is adjusted to allow a higher rate of substrate supply to the mitocondria^28^.

According to our results, some histological muscle features are typical of diving behavior group. Higher amount of IMCL are suggested to correspond with this group of animals, while significantly higher IAT and larger fiber size are described. Some of these muscular adaptations could enable cetaceans to increase their breath-hold diving by increasing substrates stores and minimizing metabolic rates.

Our results also showed the larger fibre size for any marine mammal until date, the Cuvier’s beaked whale, which perform the deepest and longest dive duration known.

## Material and Methods

### Necropsy and tissue sampling

The *longissimus dorsi* (LD) muscle of small and large odontocetes and mysticetes (n = 146) that stranded over a 12-year period (1996–2008), including 15 different species, was examined. The LD muscle is part of the epaxial musculature, which lies along both sides of the vertebral column. It is involved primarily in the cetacean’s locomotion, allowing for the upstroke in these animals^35^,^36^. The animals were of both sexes and ranged in age from neonatal to adult-old according to biological and morphometric parameters. Species included in this study are displayed in Table 1. Samples from LD muscle were collected during complete and standardized necropsies^37^. Required permission for the management of stranded cetaceans anywhere within the Canarian archipelago was issued by the environmental department of the Canary Islands’ Government. No experiments were performed on live animals due to our work is based on dead stranded cetaceans and the field studies did not involve endangered or protected species.

The conservation status was established according to Geraci and Lounsbury’s code^38^. The muscle examination included a macroscopic evaluation of the epaxial and hypaxial mass through a series of transverse and longitudinal sections separated approximately 1–2 cm throughout the entire muscle body, and a histological assessment of the LD muscle tissue from all cases. For histology, muscle samples were taken from the middle portion of the LD, lying immediately lateral to the dorsal fin, as previously described^9^,^39^.

### Histological assessment

The samples were mounted on a tongue depressor (fixed at both ends by pins) with the myofibers lengthwise-oriented and immersed in 4% neutral-buffered formalin for 24–48 hours. Transverse and longitudinal muscle sections were then carved and routinely processed, embedded in paraffin, serially sectioned and stained with haematoxylin and eosin (HE) (5 μm), periodic acid-Schiff (PAS) (4 μm) for glycogen, including amylase digestion, phosphotungstic acid haematoxylin (PTAH) (4 μm) for collagen and muscle fibers, Von Kossa (6 μm) for calcium, and osmium tetroxide (OT) postfixation technique for microscopic examination of neutral fat, as described by Abramowsky and others (1981)^30^,^40^,^41^,^42^,^43^. Briefly, formalin-fixed tissues were cut into sections no thicker than 2–3 mm to ensure adequate reagent penetration. After washing in distilled water for 30 minutes, they were placed in a closed bottle with a 1% aqueous solution of OT for 2 hours under continuous agitation. The samples were then rinsed under running tap water for 30 minutes, immersed in 0.5% periodic acid until the dark, osmicated tissues were uniformly cleared (30 minutes), and then rinsed again under running tap water. Finally, the samples were placed in distilled water and then processed routinely. This method allows full preservation of lipid and maintenance of inclusions shape and size.

The study of muscle fat infiltration consisted of the assessment of the percentage area occupied by the perimysial adipocytes. For morphometry, two groups were created: shallow diving group, comprising 5 Atlantic spotted dolphins (*Stenella frontalis*) and 5 Atlantic bottlenose dolphins (*Tursiops truncatus*); and the deep diving group, including 5 short-finned pilot whales (*Globicephala macrorhynchus*), and 5 Cuvier’s beaked whales (*Ziphius cavirostris*). For each specimen 5 randomly selected fields (including muscle fibers and perimysium) were measured at 200X magnification.

Distinguishing between pathological and merely autolysis-related morphological changes is essential in pathologic examinations. This is of particular interest in cetaceans wherein histopathological assessment is not always based upon freshly died animals. In order to evaluate and recognise autolysis-related microscopic changes and artefacts at different post-mortem intervals the following study was designed. Duplicate skeletal muscle samples were kept at room temperature and exposed to freshwater, respectively, collected and analysed at multiple time points from T_0_ (8 hours post-mortem) to T_6_ (80 hours post-mortem). This experiment was conducted on LD tissue samples from a adult male Atlantic spotted dolphin.

### Immunohistochemical study

For immunohistochemical (IHC) analyses, monoclonal mouse anti-myosin Skeletal fast M4276 and Skeletal slow M8421 antibodies (Abs) (Sigma Co., St. Louis, MO, USA) were utilized for demonstration of the fast and slow myosin heavy chain (MHC) isoforms, type II and type I, respectively. Polyclonal rabbit anti-human myoglobin (A0324) and anti-human fibrinogen (A0080) Abs (Dako, Glostrup, Denmark) were employed for Mb and fibrinogen. IHC visualization was achieved by the avidin-biotin-peroxidase method (Vector Laboratories, Burlingame, California, USA). Tissue sections in which the primary Abs were replaced by phosphate-buffered saline or nonimmune serum (rabbit or mouse) were used as negative controls to confirm the specificity of the test and no immunolabelling was observed^44^.Goat and human tissues were used as positive controls. Slow MHC recognises type I fibres (slow-twitch fibres) and fast MHC recognises type IIa, IIb and IId (IIx) (in fast-twitch fibres). Immunohistochemical identification of muscle fibre types was performed on 15 different species.

### Electron microscopy study

A subset of samples was selected for ultrastructural studies, skeletal muscle samples were fixed in 2.5% glutaraldehyde in 0.1 M sodium cacodilate buffer (pH 7.2). The specimens were postfixed in 1% osmium tetroxide in 0.2% veronal buffer, gradually dehydrated in an alcohol series, and embedded in EMbed 812 epoxy resin (Electron Microscopic Science, Hatfield, Pennsylvania, USA). Thin sections were stained with uranyl acetate and lead citrate. The sections were examined with a ZEISS EM-912 transmission electron microscope (Carl Zeiss, Oberkochen, Germany).

### Myofiber sizes comparison

A quantitative analysis was performed in six cetacean species with different diving behaviours: 4 short-beaked common dolphins (*Delphinus delphis*), 2 striped dolphins (*Stenella coeruleoalba*), 6 Atlantic spotted dolphins among the shallow diving group; and 2 pygmy sperm whales (*Kogia breviceps*), 5 Cuvier’s beaked whales, and 3 Gervais’ beaked whale (*Mesoplodon europaeus*) within the deep diving group. For morphometric analysis, adult specimens in good body condition were preferentially selected.

For morphometry, the cross sectional area (CSA) of each type of myofiber immunohistochemically stained for anti-fast myosin, was measured in ten randomly selected fields (200X magnification). Images were captured using a digital Altra20 camera (2 MegaPixel CMOS colour camera for light microscopy, Olympus Soft Imaging Solutions GmbH, Münster, Germany) and saved as TIFF for the posterior analysis (Digital software Imaging Solutions, CellA). The mean values were obtained using the Soft Imaging System for Life Science Microscopy.

Quantitative measures of the myober lesser diameter (the greatest distance between the opposite sides of the narrowest aspect of the fiber) were determined using saved digital micrographs of cross sectional muscle sections (TIFF) from the same specimens. Ten myofibers of each type were randomly selected and measured (Image Pro Premier 9.1 software). Values were reported as means ± standard errors and data were statistically analyzed using the Mann Whitney U test (SPSS v.22). The significance level was set at P < 0.05 to compare myofiber size (diameter) within groups.

### Statistical analysis

The data for each age group (shallow versus deep diving) were expressed as the mean ± standard error of the mean (SEM) and were tested for normality and homogeneity of variance using the Shapiro–Wilkes and Levene tests, respectively. The Mann Whitney U test (SPSS v.22) was used to test the statistical significance of differences in the mean values for percentage area of the interstitial adipose tissue [henceforth Intramuscular Adipose Tissue (IAT)], of the shallow and deep diving individuals. The significance level was set at P < 0.05.

## Additional Information

**How to cite this article**: Sierra, E. *et al.* Comparative histology of muscle in free ranging cetaceans: shallow versus deep diving species. *Sci. Rep.* **5**, 15909; doi: 10.1038/srep15909 (2015).

## Figures and Tables

**Table 1:** Signalment of number of animals and species included in this study and lipid content expressed as percentage of animals showing intramyofibre lipid droplets with the osmium postfixation technique.

Species	*n*	IMCL
Fin whale (*Balaenoptera physalus*)	3	0%
Short-beaked common dolphin (*Delphinus delphis*)	10	0%
Short-finned pilot whale (*Globicephala macrorhynchus*)	12	41.6%
Risso’s dolphin (*Grampus griseus*)	3	0%
Pygmy sperm whale (*Kogia breviceps*)	11	0%
Fraser’s dolphin (*Lagenodelphis hosei*)	2	0%
Blainville’s beaked whale (*Mesoplodon densirostris*)	3	66.7%
Gervais’ beaked whale (*Mesoplodon europaeus*)	6	0%
Sperm whale (*Physeter macrocephalus*)	9	44.4%
Striped dolphin (*Stenella coeruleoalba*)	25	0%
Atlantic spotted dolphin (*Stenella frontalis*)	23	0%
Spinner dolphin (*Stenella longirostris*)	2	0%
Rough-toothed dolphin (*Steno bredanensis*)	3	0%
Bottlenose dolphin (*Tursiops truncatus*)	13	0%
Cuvier’s beaked whale (*Ziphius cavirostris*)	19	52.6%

**Table 2:** Fibre size data (CSA and LD) for each species from two groups with different dive behaviour; shallow (*D. delphis*, *n* = 4; *S. coruleoalba*, *n* = 2; and *S. frontalis*, *n* = 6) *versus* deep-diving species (*K. breviceps*, *n* = 2; *M. europaeus*, *n* = 3; and *Z. cavirostris*, *n* = 5^*^).

Group	CSA (μ^2^)		LD (μ)		
	Type I	Type II	Type I	Type II	II/I
Shallow-diving	661,94 ± 129,91	1709,36 ± 124,29	33,02 ± 1,35^*^	55,84 ± 6,26^**^	1,7
*D. delphis*	640,15 ± 254,52	1586,48 ± 606,47	31,58 ± 4,11	50,83 ± 2,10	1,6
*S. coeruleoalba*	544,30 ± 62,82	1706,59 ± 4,39	34,24 ± 1,36	62,85 ± 0,48	1,83
*S. frontalis*	801,37 ± 394,39	1835,02 ± 580,52	33,24 ± 5,08	53,83 ± 5,62	1,62
Deep-diving	2242,19 ± 1721,19	4690,46 ± 2284,00	51,93 ± 22,67^*^	83.95 ± 18,97^**^	1,61
*K. breviceps*	4033,98 ± 1020,81	3491,08 ± 470,78	74,39 ± 0,77	72,62 ± 5,65	0,97
*M. europaeus*	601,58 ± 319,46	3256,00 ± 1115,14	29,08 ± 3,33	73,38 ± 8,03	2,52
*Z. cavirostris*	2091,00 ± 1444,68	7324,31 ± 2877,30	52,35 ± 11,87	105,84 ± 17,43	2,02

Values are expressed as mean (±s.d.). An extra specimen was added within the *Z. cavirostris* species in the morphometric analysis for the LD in order to homogenise the number of both groups. The Mann-Whitney U test for two independent-samples revealed a highly significant difference in the mean LD of the type I^*^ (P = 0.019) and type II^**^ (P = 0.000) myofibers when shallow- and deep-diving groups were compared.

**Table 3:** The area occupied by the adipocytes intermixed within the perimysium was show as the percentage with respect to the total sample area.

Group	%IAT
Shallow-diving	1,95 ± 2,10
*S. frontalis (n *=* 5)*	3,44 ± 3,44
*T. truncatus (n *=* 5)*	0,47 ± 0,41
Deep-diving	26,01 ± 1,03
*G. macrorhynchus (n *=* 5)*	26,74 ± 12,24
*Z. cavirostris (n *=* 5)*	25,29 ± 12,39

Values are expressed as mean (±s.d.). A statistically significant (P = 0.000) difference was obtained when both groups were compared by the Mann-Whitney U test for two independent-samples.
